# The Athlete’s Heart and Machine Learning: A Review of Current Implementations and Gaps for Future Research

**DOI:** 10.3390/jcdd9110382

**Published:** 2022-11-08

**Authors:** Ryan A. A. Bellfield, Sandra Ortega-Martorell, Gregory Y. H. Lip, David Oxborough, Ivan Olier

**Affiliations:** 1 School of Computer Science and Mathematics, Liverpool John Moores University, Byrom Street, Liverpool L3 3AF, UK; 2 Liverpool Centre for Cardiovascular Science at University of Liverpool, Liverpool John Moores University and Liverpool Heart & Chest Hospital, Liverpool L3 3AF, UK; 3 Department of Cardiology, Liverpool Heart & Chest Hospital, Liverpool, L14 3PE, UK; 4 School of Sport and Exercise Sciences, Liverpool John Moores University, Byrom Street, Liverpool L3 3AF, UK

**Keywords:** athlete’s heart, cardiology, machine learning, electrocardiography, echocardiography, pre-participation screening

## Abstract

Background: Intense training exercise regimes cause physiological changes within the heart to help cope with the increased stress, known as the “athlete’s heart”. These changes can mask pathological changes, making them harder to diagnose and increasing the risk of an adverse cardiac outcome. Aim: This paper reviews which machine learning techniques (ML) are being used within athlete’s heart research and how they are being implemented, as well as assesses the uptake of these techniques within this area of research. Methods: Searches were carried out on the Scopus and PubMed online datasets and a scoping review was conducted on the studies which were identified. Results: Twenty-eight studies were included within the review, with ML being directly referenced within 16 (57%). A total of 12 different techniques were used, with the most popular being artificial neural networks and the most common implementation being to perform classification tasks. The review also highlighted the subgroups of interest: predictive modelling, reviews, and wearables, with most of the studies being attributed to the predictive modelling subgroup. The most common type of data used was the electrocardiogram (ECG), with echocardiograms being used the second most often. Conclusion: The results show that over the last 11 years, there has been a growing desire of leveraging ML techniques to help further the understanding of the athlete’s heart, whether it be by expanding the knowledge of the physiological changes or by improving the accuracies of models to help improve the treatments and disease management.

## 1. Introduction

Heart disease is the leading cause of death worldwide, accounting for 16% of the total world’s deaths in 2019 [1]. In the UK alone, around 7.6 million people are living with heart disease which causes, on average, one death every three minutes [2]. Exercise is one of the best methods for improving health and reducing the cardiovascular risk factors [3]. However, extreme exercise regimes, such as those followed by athletes, cause physiological changes in the heart to help it cope with the increased demands placed upon it [4]. These physiological changes, also known as the “athlete’s heart”, can cause issues as they are difficult to distinguish from pathological changes, exposing athletes to sudden cardiac death [4].

Sudden cardiac death is the most common cause of death in young athletes, with current estimates placing its incidence rate between 1 in 40,000 and 1 in 80,000 athletes per year [5]. To prevent this, pre-participation screening, using techniques such as electrocardiography (ECG) and echocardiography, are used to identify the cardiovascular conditions associated with a sudden cardiac death, allowing for appropriate treatments and avoiding adverse outcomes. Although shown to be generally effective, there are still approximately 1% false positives, resulting in some athletes going undiagnosed, e.g., the cardiac arrest of Christian Eriksen at the Euro 2020 tournament and Fabrice Muamba in the FA Cup quarterfinals in 2012).

Artificial intelligence (AI) has rapidly grown over the last decade, with machine learning (ML) accounting for the majority of this growth [6]. ML techniques, powered by advances in computational performance and very large datasets, have shown a great success and they frequently outperform human performance [7]. ML is commonly used in supervised and unsupervised learning tasks. Supervised ML techniques work in two parts: first, the ML algorithm is trained using input variables and labelled output variables to learn the associates between the two, then, the trained model is used to make predictions on a test set, again where the labels of the outputs are known, to assess the performance [8]. Some examples of these methods include artificial neural networks (ANN), Random Forest, etc. Unlike supervised ML, unsupervised ML uses unlabelled data and automatically finds the key relationships and structures within the data. Two examples of such methodologies are t-distributed stochastic neighbourhood embedding (t-SNE) and principal component analysis (PCA).

The use of ML techniques applied to diagnostic investigations may prove valuable to help detect cardiac conditions in athletes, establish the risk levels, and develop an understanding of the physiological changes more accurately. ML models trained using different data modalities and data formats have been applied successfully in detecting many cardiovascular issues [9,10,11,12,13], showing how ML can solve a range of tasks, such as predicting mortality following a cardiac intervention [13], in specific populations of individuals [12], predicting coronary heart disease [10] and estimating the prognosis of patients with congenital heart failure [11]. 

The aim of our study, therefore, is to review the current state of ML applied to the athlete’s heart by evaluating the current trends regarding the ML methodologies and approaches used within the area and determining the relevant questions and problems ML currently faces. To this end, we plan to focus the review on the following: (1) ML applications in the assessment of the athlete’s heart, and (2) understanding the desire to implement ML approaches within this area of research.

## 2. Methods

### 2.1. Search Strategy and Selection Process

To obtain the data needed to carry out the review, the Scopus and PubMed online electronic databases were searched to return the relevant literature. Table 1 outlines the criteria used to define the search term and where, within the manuscript, each term focuses. The literature returned from the searches was then reviewed and filtered by two authors, RAAB and DLO, by the titles and abstracts, and then through full-text readings, which were carried out by RAAB, so that only the studies relevant to the review were included.

### 2.2. Search Results

**Figure 1:**
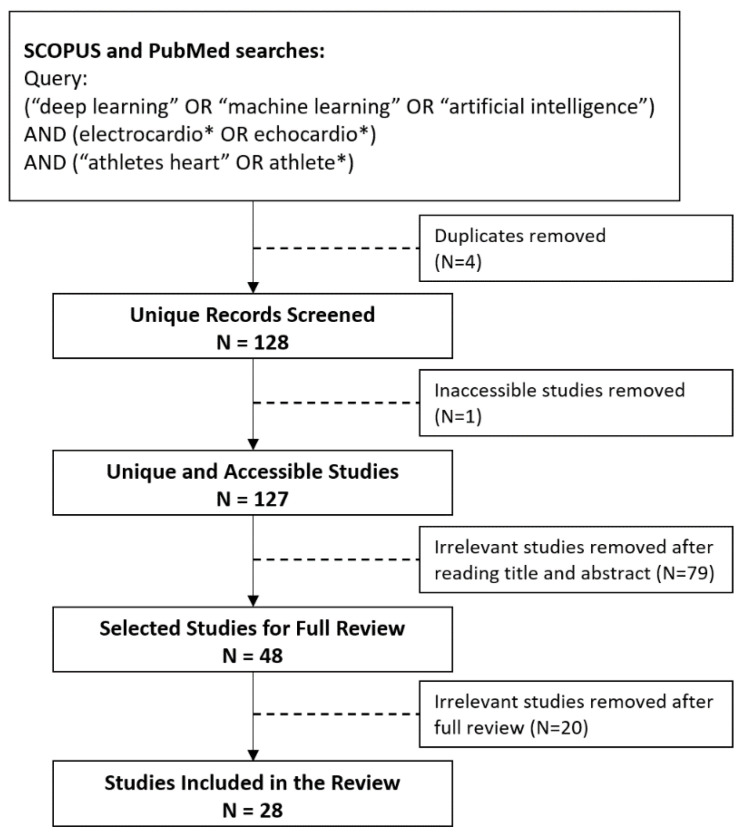
Study selection flow chart.

The search process is detailed in Figure 1. Based on the search criteria, 132 total studies were returned from the searches performed on the Scopus and PubMed online databases. The unique studies from these searches were subsequently extracted, which left a total of 128 studies. The titles and abstracts of these 128 studies were reviewed, resulting in 79 studies being excluded as they were deemed to be not relevant due to having a different focus area than the one specified for this review.

Of the 49 studies that remained, 1 study was excluded from the review due to issues with accessing the full manuscript, leaving 48 studies to be included for full-text readings and to form the dataset for this review. However, during the full-text readings, a further 20 studies were excluded: 16 were excluded as they were deemed to be not relevant to the review, and the other 4 were excluded due to concerns about their quality, i.e., being vague and having an unclear description of either their methodology or approach used to develop their models, how the evaluation criteria were presented, and why certain metrics were used over others. After all the exclusions had been applied, this left a final total of 28 studies that were considered for this review [4,14,15,16,17,18,19,20,21,22,23,24,25,26,27,28,29,30,31,32,33,34,35,36,37,38,39,40]. 

## 3. Results

### 3.1. Study Subgroups

Of the 28 studies, several different approaches were taken. We clustered the studies into four subgroups: predictive modelling, reviews, wearables and others. Each study was then assigned to one of these four groups using the criteria outlined in Table 2. “Predictive Modelling” made up most of the studies with 10 (36%) [14,16,21,22,26,27,31,32,34,40] being assigned to this group. “Reviews” was the next single largest group with eight (29%) studies [15,17,18,23,29,30,35,38]. “Wearables” was the smallest single group with four (14%) studies [20,25,28,37]. The final six (21%) unassigned studies [4,19,24,33,36,39] were placed in the “Others” group as they did not meet the inclusion criteria for the previous groups. 

#### 3.1.1. Predictive Modelling

The studies within this group are focused on using methods that can be applied to a dataset to attribute one of two or more classes to each patient or participant. This has been approached in two main ways. The first and most popular type of approach implemented was to use ML to learn from the data and make predictions on what class each patient/participant should be classified as automatically. Eight of the studies [14,16,21,22,26,27,34,40] use this approach, applying ML algorithms in varying levels of complexity, from linear discriminant analysis (LDA) to ANN. A more in-depth discussion of the individual methods which were used and their respective applications can be found in the section “Machine learning approaches used”.

The second approach used in the other two studies forgoes the use of ML and instead focuses on defining an algorithm tree that can be manually followed by a human user to help improve the accuracy of their diagnoses. Vergani et al. [31] proposed a diagnostic algorithm that can be used by healthcare professionals to distinguish between a hypertrabeculation phenotype, noncompaction phenotype, and left ventricular noncompaction cardiomyopathy. Viviers et al. [32] focused on comparing the predictions made by a sports physician using a history questionnaire and a physical examination, to a technician using computer-assisted auscultation on the nature of cardiac murmurs in collegiate athletes. These two approaches are focused on classification, as with the ML-focused studies, but they have done so in a way that only utilizes human expertise.

#### 3.1.2. Reviews

Within the data, there were eight studies which were classified as reviews. Georgijević and Andrić [17] and Lucas et al. [23] had relatively similar aims: they both reviewed the current use of different modalities in the pre-participation screening of athletes, with Georgijević and Andrić [17] looking specifically at ECG and Lucas et al. [23] concentrating on echocardiography. These studies also review the guidelines for how their respective modality should be used in the pre-participation environment and the benefits that they provide. Higgins et al. [18] had a different focus and instead reviewed the different defects that can cause a sudden cardiac death in young athletes and recommend which modalities are best suited to best diagnose each. Chang [38] also focused on the ECG, but their approach was to consider the positives and negatives of applying it to screening young adults, as well as a brief discussion on how AI is likely to shape the future of the heart screening of athletes. Conversely to the studies already mentioned, Beavers and Chung [35] and Seshadri et al. [29] both centred their reviews on wearables. More specifically, Beavers and Chung [35] highlighted the emerging wearable technologies and how they can be used to aid in heart assessments, with specific examples focused on minimising the cardiovascular risk in athletes. Seshadri et al. [29] reviewed the ways in which the data collected from wearables had been analysed with ML to evaluate athletes’ heart health, with several successful implementations reported to have achieved accuracies as high as 98% in the prediction of obstructive hypertrophic cardiomyopathy.

The remaining two studies are systematic reviews: Claudino et al. [15] focused on the sports performance and injury risk of athletes within team sports and highlighted which AI techniques have been applied within each sport, while Van Eetvelde et al. [30] looked more specifically at the ML methods which have been used in the prediction and prevention of general sports injuries. Our review differs from both Claudino et al. [15] and Van Eetvelde et al. [30] in two key areas: (1) we focus on highlighting ML applications towards the athlete’s heart exclusively, instead of the wider research area of injury prevention and risk, and (2) we aim for a more comprehensive overview of the ML approaches themselves, and emphasise the relevant challenges that are present and how to address them through future research. 

#### 3.1.3. Wearables

The four studies in this category share the same goal: they describe the development or implementation of wearable hardware that can be used by athletes to help collect physiological data automatically. However, they differ in their individual implementations of the wearable technology, and in how the data are collected, stored, and analysed. Adetiba et al. [25] developed a smart jersey to be worn by athletes to automatically record an ECG signal. These data are then automatically passed through an ANN that has been pre-trained to identify heart defects and returns whether the result is normal or not to a smartphone application. Hussain et al. [20] proposed a fog-centric, wireless, and real-time framework for health and fitness analysis, which consists of collecting data such as ECG recordings, body movement, and posture from multiple wearables simultaneously, which is then fed into two ML models: one to predict the exercise being performed by the athlete; the other to predict the athlete’s health state. Similar to the aforementioned studies, Castillo-Atoche et al. [37] described the development of a new wearable ECG with a dynamic power management strategy that then automatically passes the collected data to an ML model to detect arrhythmias in real time. Unlike Adetiba et al. [25] and Hussain et al. [20], the final study in this group by Rymarczyk et al. [28] concentrated exclusively on the development of a new type of electrode for physiological signal sensing as an alternative to a conventional gelled electrode.

#### 3.1.4. Others

The remaining six studies do not match any of the criteria for the three main groups. Instead, these are individual pieces of research that provide a different overview of the athlete’s heart. Chatzakis et al. [39] focused on developing an electronic health record, with a built-in decision support system, to support paediatric cardiovascular disease screening. Dockerill et al. [33] utilised a case series approach to assess the hearts of 27 runners before and after an extreme running event whilst documenting the changes in the cardiac structure caused by an acute bout of exercise. Similarly, Kerkhof et al. [4] investigated the changes in the heart of a select group: three division one undergraduate crew athletes explored the use of ‘focused’ echocardiography in screening athletes to assess their heart health and function.

The studies by Bernardino et al. [36], Huang et al. [19], and Mlynczak and Krysztofiak [24] bring unique approaches. Bernardino et al. [36] used cardiac magnetic resonance imaging data for athletes and non-athletes and applied several techniques, such as statistical shape analysis and dimensionality reduction, to highlight the areas of the heart that underwent a remodelling due to endurance exercise (more details on the methods used are discussed in the section on the “Machine learning approaches used”). Huang et al. [19] is the only study to leverage unsupervised clustering to investigate the validity of sport-specific adaptions in athletes’ hearts (the methods are further discussed in the section on the “Machine learning approaches used”). Mlynczak and Krysztofiak [24] focused on discovering causal relationships between cardiovascular and respiratory variables in elite athletes whilst they were supine and standing, aimed at developing appropriate training plans.

### 3.2. Data Modalities Used for Athlete’s Heart Assessment

**Figure 2:**
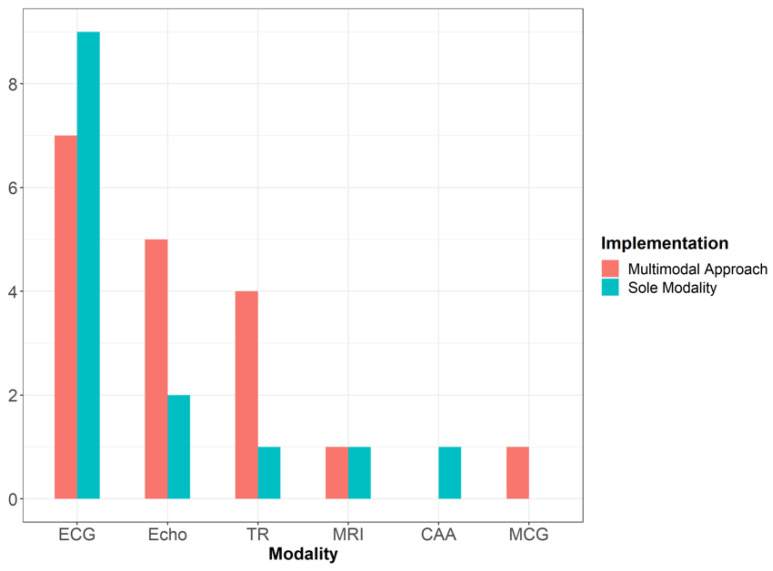
Displays the number of times each modality was mentioned within the studies. It also displays how often the modality was implemented on its own, or in conjunction with another modality. Abbreviations: ECG = electrocardiogram, Echo = echocardiogram, TR = tabular records, MRI = cardiac magnetic resonance imaging, CAA = computer-assisted auscultation, MCG = magnetocardiogram.

Within this review, a data modality refers to the type of data collected. There are various modalities mentioned within the studies being reviewed, from images to signal data. There are examples of these being used as a sole modality as well as examples where information from multiple modalities have been used to evaluate the heart, with the splits for all the modalities mentioned displayed in Figure 2.

Our review highlighted that only 23 of the 28 studies mentioned which modality, or a combination of modalities, were used to either review or generate their dataset. The most commonly used was an ECG, with it listed in 16 of the studies, and it is the sole modality used in 9 of the studies. This is expected due to it being able to detect several conditions associated with sudden cardiac death in athletes, such as hypertrophic cardiomyopathy, arrhythmogenic right ventricular cardiomyopathy, myocarditis, dilated cardiomyopathy, brigade syndrome, long QT syndrome, and Wolff-Parkinson-White syndrome [18]. The use of the ECG as part of athletes’ screening is recommended by associations worldwide, including the European Society of Cardiology (ESC) and the International Olympic Committee, highlighting its widespread application within the literature [17]. ECGs are also very commonly used among healthcare practitioners due to them being a cost-effective, non-invasive technique with a relatively high sensitivity for detecting underlying cardiac disease [23].

Echocardiography is the next most commonly used modality, with it being used in conjunction with other techniques in seven studies, with it being the sole modality used in two. Like with an ECG, echocardiography is widely used for many of the same reasons. It is non-invasive and, compared with other imaging modalities such as CT imaging and MRI, it is cost-effective [18]. It also plays a crucial role in diagnosing some conditions where the ECG is less sensitive such as Marfan Syndrome, coronary anomalies, and dilated cardiomyopathy [18]. Echocardiography also yields positive results when used in conjunction with the information generated from other sources, such as ECGs, in a multimodal approach [23].

Another popular modality that has seen some use is the tabular records, which encapsulates various sources of information relating to the patient/participant such as their age, sex, and race. This modality was referred to in five studies, with it appearing as the sole modality once. Rahman et al. [27] gave a compelling reason as to why tabular records should not be used as a sole modality in regard to the evaluation of athletes’ hearts. Their use of the tabular information taken from the American Heart Association questionnaire for classification was not able to perform as well as a cardiologist that had both ECG and echocardiographic data available. However, it does serve an important purpose, as certain demographics such as age, race, and gender have already been shown to affect the heart differently, so ignoring this information may lead to overlooking a key insight. This point is further supported by Narula et al. [26], whereby using information derived from both tabular records and the echocardiogram, they built an accurate predictive model (the specific model performances, with metrics, can be found in the section on the “Machine learning approaches used”).

Other modalities are referenced; however, they are used less frequently than the three most popular modalities: electrocardiography, echocardiography, and tabular records, discussed above. Cardiac MRI is referenced twice [31,36], and computer-assisted auscultation [32] and magnetocardiography [22] are both mentioned once. The reasons for this trend likely lie in the already highlighted cost-effectiveness and non-invasive nature of the three popular modalities when compared to their alternatives.

A common theme throughout the studies is that in the majority of cases, the key features pre-extracted from the modality are analysed instead of the raw data itself. The features can either be extracted manually by a healthcare professional, such as the physical measurements [21,22,26], or by using a technique to generate statistical features instead [14,40]. The only study that bucks this trend was by Castillo-Atoche et al. [37], where they developed their model on ECGs in an image format instead.

### 3.3. Machine Learning Approaches Used

The application of ML has been used in 13 of the studies considered for this review. Eight of them were assigned to the “predictive modelling” group, three were assigned to the “wearables” group, and two were assigned to the “other” group. The most commonly used method was the ANN, with it being used in 5 out of the 13 studies [14,21,25,26,40]. This was then closely followed by support vector machines [21,26,27,40], used in 4 out of the 13 studies, and then random forest [26,27,40] and logistic regression [16,34,36], tested in 3 out of the 13 studies. Other techniques that were also mentioned within the literature but were less commonly used were decision trees [16,34], naïve Bayes classifiers [21,27], multiple linear regression [19], k nearest neighbours [21], linear discriminant analysis [22] and long-term short memory neural networks (LSTM) [20], convolutional neural networks (CNN) [37], and hierarchical clustering [19]. A summary of all 13 studies can be found in Table 3 which details the aims of each study along with other key information.

The main application of ML within these 13 studies is towards classifying whether a patient/participant has a particular heart disease or defect, with 8 out of the 13 having this focus. Adetiba et al. [14] used an artificial neural network to classify whether an athlete’s heart is normal, or whether one of the following defects was present: tachyarrhythmia, bradyarrhythmia, or hypertrophic cardiomyopathy. This was done by extracting the ECG signals, applying a first-order statistical signal processing technique, and passing these features as inputs to train the model. The final model reported an accuracy of 90%. A subsequent study [25] from the same authors, published two years later, performed the same classification task, included feature extraction methods, and used only ANN. However, this time the data were generated by a wearable jersey they designed, reporting an accuracy of 100%.

Lombardi et al. [22] used linear discriminant analysis to determine whether patients with idiopathic ventricular arrhythmias with a left bundle branch block and inferior axis morphology arrhythmia originated from the aortic sinus cusps or the right ventricular outflow tract. Manually extracted features from multiple modalities were used to create the linear separation between the two classes, achieving a final accuracy of 94.7%. The aim of Narula et al. [26] was to discriminate between the hypertrophic cardiomyopathy and physiological hypertrophy in athletes. The manually extracted features from the echocardiographic scans as well as tabular records were used as the inputs to train a support vector machine, random forests, and an ANN model. The predictions from each model were taken and a voting system was used to determine the overall class of the patient. The reported performance of this ensemble method was an AUC of 0.795.

Długosz et al. [16] used different ML techniques in an attempt to address the two aims of the study, which were to use ECGs to estimate the level of cardiac troponin (cTnI) in amateur athletes as well as detect coronary artery disease (CAD) in the same cohort of patients. The cTnI levels of the athletes were recorded at several times before and after a sporting event, and CAD was confirmed in six athletes. The study attempted (unsuccessfully) to train a logistic regression model to estimate the cTnI levels. However, they were able to detect CAD successfully by training a grid search optimised decision tree using the pre-extracted features from ECGs performed on the athletes and tabular records such as their BMI and age and the blood levels of the cTnI. The best performing model achieved an AUC of 0.91.

The work by Rahman et al. [27] differs from the above three studies as it forwent any formal screening test data such as ECGs or echocardiograms and used the tabular record information collected from the American Heart Association questionnaire. It aimed to predict whether an athlete’s heart was normal or not and it did this by training three models: a support vector machine, a random forest, and a naïve Bayes classifier. They performed two experiments, the first was on the whole dataset, which contained a large positive class (representing healthy hearts) bias, and another on a dataset where the positive class had been subsampled to create a biased dataset. The best results reported for these experiments were an accuracy of 0.742 using the support vector machine for the first experiment, and 0.553 using the random forest for the second experiment.

Regardless of their stated results and methodology, many of the studies referred to previously share a similar drawback: they all used a small dataset for their analyses. The size of the dataset used by Adetiba et al. [14,25] is n = 40, Lombardi et al. [22] is n = 26, with Narula et al. [26], Długosz et al. [16], and Rahman et al. [27] using larger datasets of n = 139, n = 160, and n = 470 participants, respectively. The use of small datasets can lead to problems when trying to leverage ML methods such as ANN, whereby the model will not learn the underlying relationship between the input variables and the output, potentially resulting in the model overfitting the data and reducing its ability to generalise to new, unseen data. Barbieri et al. [34] and Castillo-Atoche et al. [37] both addressed this issue by using much larger datasets for their analysis. Barbieri et al. [34] used 26,002 participants for their analysis, to classify whether an athlete is at a cardiovascular risk or not. For this, the authors use tabular record information as well as the features manually extracted from ECGs as inputs to train and test two models, one built using a decision tree, the other using logistic regression. The logistic regression model provided the best performance, generating an AUC of 0.78. Castillo-Atoche et al. [37] used a much larger dataset consisting of 56,542 ECG samples taken from 487 patients to automatically predict arrhythmias in athletes in real time. The ECG samples were analysed in an image format, with 55,222 samples taken from 480 subjects used for training and 1320 samples taken from 7 athletes used for the test. The training dataset was pieced together using several open-access online datasets, with the test set comprised of a manual reading taken from their discussed wearable. The model used to make the predictions was developed using a CNN and achieved an accuracy of 94.3% on the training set and an average accuracy across the seven athletes in the test set of 93.9%.

The remaining five studies that applied ML techniques have a different focus other than disease classification. Christ and Rückert [40] aimed to use ML to predict whether a participant was an athlete or not based on their ECG criteria. The authors used statistical measurements for time-domain features and discrete Fourier transforms to extract the frequency domain features that were then used as model inputs. An ANN, a support vector machine, and a random forest model was trained and tested on the data, with the best performance coming from the random forest model which generated an accuracy of 98.1%. 

Laurino et al. [21] focused on classifying the heart states in athletes, distinguishing between heart rates that were at rest and those during stressful conditions. Like with many of the approaches stated thus far, the features from the ECGs were manually extracted to be used as the dataset for this analysis. K nearest neighbours, support vector machines, naïve Bayes, and artificial neural networks were all tested, and the best result came from the artificial neural network, which successfully managed to separate the two classes with an accuracy of 0.87 and 0.86 on the training and test set, respectively.

Hussain et al. also used a similar application of ML [20] whereby they used an LSTM neural network on the waveforms generated from the heart rate, breathing rate, and heart rate variability, to predict the athletes’ health state. The health state considered for the analysis were aerobic, anaerobic, V02 max, hazardous, and moderate, and their model was able to classify the athletes with an accuracy of over 97%. Hussain et al. [20] also described a second ML application, where they again used an LSTM network to predict what activity the athlete was performing. They trained four models for four different experiments, all using breathing waveform data and the ECG data as the inputs, with the best stated predictive performance being an accuracy of over 83%.

Huang et al. [19] are different from the former as they leveraged unsupervised learning in an attempt to find hidden clusters within the dataset. The study had two aims: (1) to explore the natural clustering of echocardiographic variables to identify athlete groups with similar characteristics; and (2) to investigate the validity of sport-specific adaption through a data-driven approach for evaluating the athlete’s heart. To address the first aim, through utilising standard statistical tests such as an ANOVA and *t*-tests as well as multiple regression analysis, they were able to show clear training-related adaptations between the groups which were defined by using Mitchell’s classification. For the second aim, the agglomerative hierarchical clustering managed to find two distinct clusters for both male and female athletes, confirming sport-specific adaptions.

The final study by Bernardino et al. [36] used a different approach and ML implementation to the other twelve studies. They presented a linear statistical shape analysis framework that looked for shape differences between the athletes and a set of control participants. This framework works by using a combination of dimensionality reduction techniques, principal component analysis, and partial least squares to reduce the high dimensional shape vectors to a latent space that contains the most relevant shape patterns. Logistic regression was then used to classify what shape patterns were the most discriminating between the two populations, and then they used this information to provide a visual representation of the changes. This framework was applied to cardiac magnetic resonance imaging for the study population which was able to highlight areas of the heart that undergo a cardiac remodelling due to endurance exercise.

There is a total of 11 years between the earliest study published by Laurino et al. [17] in 2011 and the most recent study published by Castillo-Atoche et al. [37] in 2022. Over most of this time, the implementation of machine learning was fairly straightforward: selecting a classification task, testing several techniques to find which performed the best, and reporting the results. However, more recently, the types of ML techniques which have been used have become more complex and intricate, as seen in Hussain et al. [20] being the first to leverage deep learning methodologies in the form of an LSTM, and Castillo-Atoche et al. [37] leveraging the power of CNNs for image analysis. Additionally, the problems ML are being applied to are becoming more focused and novel, as seen in Bernardino et al. [34] and Huang et al. [19]. This indicates the beginning of a trend towards a more in-depth ML analysis being implemented within the research area.

## 4. Discussion

The studies evaluated as part of this review indicate that there is a clear drive within the research area of the athlete’s heart to leverage ML. This is shown by 57% of the 28 studies either using ML to create a model to answer a question or solve a particular problem [14,16,19,20,21,22,25,26,27,34,36,37,40], or to evaluate how ML is being implemented in similar areas through review studies [15,29,30]. The most popular application of ML is in its use to generate models for classifying patients/participants to aid in diagnosing heart defects at an early stage. 

The results stated in the research are very positive, showing the real benefit ML could have should it see a widespread adoption. What the studies also show is that alongside the traditional disease and heart health predictive modelling, there is also a desire to use ML to help further develop the knowledge surrounding the athlete’s heart itself. This has been done by studies aimed at quantifying the magnitude of exercise volume on cardiac adaptations within athletes’ hearts when compared to that of the general population.

## 5. Limitations of Current Research

The use of ML is desirable in many tasks, including health care, as properly trained models can help reduce errors in diagnosis by either matching human performance [41] or even being superior in some cases [7]. Even though the ML applications in this area have shown promise, several issues could potentially slow the adoption of such techniques and limit their application in the real world. First, the vast majority of the data used in the studies that reference ML, or any of the 28 studies in the full literature reviewed, do not use an open-source dataset. This is problematic, making it difficult for external groups to assess the data to determine potential biases that were missed in the study or to validate the stated models being presented. This will likely be a difficult problem to overcome, due to the nature of the data being analysed. For the teams elite athletes compete for, having information about their players’ health, or obtaining it for elite athletes from other teams, can give an unfair advantage in situations such as transfer markets [42]. Therefore, it is not in the best interests of the main collectors of athletes’ data to make it publicly available and performing the data collection at the scale needed by third parties would become very costly. The only study that uses open-source data is by Castillo-Atoche et al. [37], where they fuse several open-source databases to form a training dataset. The datasets used are the MIT-BIH Arrhythmia Database [43], ECG-ID Database [44], MIT-BIH Supraventricular Arrhythmia Database [45], MIT-BIH Atrial Fibrillation Database [46], QT Database [47], and Long Term ST Database [48] which are all hosted on PhysioNet [49]. Even though the use of open-source data is positive, this approach does not solve the issues discussed. First, there is not a clear description of how the data has been fused and pre-processed, hampering validation efforts. Additionally, the open-source data used does not contain the athlete’s data used in the study, again further hindering the ability for an external validation.

Another issue relating to the data is that many of the studies use small sample sizes for their analyses. This poses a problem, especially with ML applications, as it is well known that having more data available can not only increase the model’s performance but increase the generalisability of the model. Adetiba et al. [25] is an example where a classic symptom of overfitting is present, as the stated accuracy is unusually high at 100%. This, paired with the very small data size and the inability to reproduce the work due to non-public data, further supports the idea that overfitting may be present in this model. Furthermore, heart defects in athletes are generally at a low prevalence in a given population, hence a small sample size is unlikely to be fully representative of the disease which the authors are attempting to analyse. Hussain et al. [20] further demonstrated the effects imbalanced data can have on the results in the accuracy stated for the health state predictions of 97%. Even though the dataset is large, the prevalence for the class of concern, whether the health state is currently hazardous, represents only 0.085% of the dataset, with the classes aerobic and moderate accounting for 92.7% of the dataset. This causes an issue as it becomes very easy for a model to overfit and generate good performance metrics by mainly predicting the majority classes. This increases the difficulty for an ML model to fully understand trends that distinguish what separates the class of interest and reduces the likelihood of it generalising well to an unseen dataset. 

As briefly mentioned previously, the analyses are mainly performed using the features extracted from the different modalities, such as electrocardiograms, as inputs for their analysis instead of the raw input itself. With the successes seen by using the raw data as inputs to develop ML models for the prediction of different heart conditions [50,51,52], it is surprising that none of the studies has attempted to implement this approach toward the athlete’s heart. Additionally, restricting the input data to the pre-extracted features only means working under the assumption that the features themselves explain enough variance between the different output classes to enable accurate predictions, which may not hold true. Another potential issue arises due to either the time and associated cost of a healthcare practitioner extracting these features manually, which can further exacerbate the small dataset issue, or using a feature extraction technique which may not fully capture all the relevant features of the original input, harming the model’s performance.

A further point here is that most models which have been built have used supervised ML as the basis of the analysis. The difficulty here again is that the data are required to be labelled for supervised ML to be carried out, meaning an expert practitioner will need to analyse the data to provide an appropriate diagnosis or status to each sample, which can be costly and time-consuming. There may also be a situation where assigning labels to the data is not appropriate or even possible to do accurately, for example where a cross-sectional study was performed with no specific outcome in mind, or if the equipment needed for a gold standard diagnosis is unavailable. This problem will only be worsened by the ever-increasing volume of the data generated and could result in large numbers of datasets being underutilized, again exacerbating the issues surrounding the lack of open-source data and small sample sizes. Another problem supervised ML has in this context is, as described in the previous paragraph, the low prevalence of adverse outcomes in the athlete’s heart. Having limited information on non-healthy hearts will likely impact the ability of any supervised ML to properly model the underlying structures that distinguish a healthy and non-healthy heart. There are techniques that can be applied to help improve the performance on imbalanced datasets, however, again, these come with their own challenges, such as potentially introducing an additional bias to the results. Considering the numerous challenges associated with supervised ML in this area, it hints that a different approach may be appropriate to generate an optimal output.

## 6. Future Research and Impact

A great first step would be an organised effort to generate large, open-source datasets consisting of athletes’ hearts data so that ML models can be built, tested, and validated by external researchers to confirm the performances of different models. This should also help in building up the trust between those developing the models and those that will be using them, which in turn may help speed up the adoption. This approach is not novel, with the creation of public databases playing a pivotal role in pushing key areas of research in closely related disciplines, such as atrial fibrillation detection [6].

A further area for future research will be to focus on applying ML models to the raw data instead of using pre-extracted features. This will have obvious benefits which have already been stated of saving time and money, in theory allowing the scope of future projects to be more ambitious. The main reason for this approach, however, is the potential for the discovery of novel biomarkers by the ML model, finding associations between the features in the raw data and the previously unknown outcome. These discoveries would help push this research field forwards, helping to strengthen the understanding of the athlete’s heart. Castillo-Atoche et al. [37] is the only study within the review that embraces the raw data in the form of ECG images. Their study clearly shows the benefits of this approach with their model being able to carry out analysis automatically and to a high degree of accuracy.

In addition to this, there should also be a focus on developing frameworks that can use ML models that can analyse the raw data from several modalities simultaneously to make its decisions. Rahman et al. [27] suggested that the data from electrocardiography and echocardiography should be considered by healthcare professionals when performing athletes’ heart screenings to yield the best results. Therefore, it seems a logical next step to evaluate whether this hypothesis transfers to ML models and if it yields tangible improvements to the model’s performance.

Another potential avenue that could be pursued is to look at developing models to determine disease progression alongside the physiological adaptations of the athlete’s heart. All the predictive modelling conducted in the above literature centres on determining the presence of the disease, not necessarily the severity of the disease or how it will develop within the subject. By expanding the research in this area, it will provide healthcare professionals with the tools and information needed to help properly manage the disease and provide the appropriate treatments earlier.

Finally, future research should start to focus on expanding the implementation of unsupervised ML due to its advantages in certain situations over supervised ML. As unsupervised learning does not require labelled data, instead finding key relationships within the data automatically, it provides a solution to the issues with datasets which were mentioned above, relating to the time and cost of labelling, as well as the data where labels are simply not appropriate. A more significant benefit of unsupervised ML in this context is that it allows for a rephrasing of the problem and provides an alternative look into the data. For example, instead of taking the classical approach and phrasing the problem as a binary classification problem, such as is the athlete’s heart healthy or not healthy, the problem can instead be constructed as an anomaly detection task and answer “What does a healthy athlete’s heart look like?”. This approach provides compelling solutions to the issues discussed in the limitations section surrounding the low prevalence of adverse outcomes in athletes’ hearts, as only healthy data would be required to develop such a model, providing solutions which give a deeper understanding of the raw data itself, as well as looking at to what degree the data are similar.

## 7. Conclusions

This review shows that there is a clear desire for using ML within the assessment of the athlete’s heart. The most commonly used ML methodologies within this research area were ANNs, support vector machines, and random forests, where the most common implementation was to perform predictive modelling in the form of disease classification. With a continued development and sustained advancements, the future potential of ML applications is promising, not only in improving model prediction accuracies, but in aiding in the understanding of the underlying physiological changes within an athlete’s heart.

## Figures and Tables

**Table 1:** Criteria used to build the literature search.

Criteria		Term Location
A	“deep learning” OR “machine learning” OR “artificial intelligence”	Anywhere within the manuscript
B	electrocardio* OR echocardio*	Anywhere within the manuscript
C	“athletes heart” OR “athlete*”	Title, Abstract, or Keywords

**Table 2:** Criteria for classification.

Group		Criteria
1	Predictive Modelling	Main aim is to use some methodology to create a model or framework that can be used to classify data
2	Review	Consolidate existing literature in some way to construct practical guidelines or conduct a systematic review, etc.
3	Wearables	Main aim is the discussion or development of wearable technology for use as either a solely data collection enterprise or to conduct automatic analysis
4	Others	Does not fit the above criteria

**Table 3:** Summary of studies that applied ML methods.

Study	Sample Size (N)	Type of Method	Problem Addressed	Performance Metrics Stated
Adetiba et al. [14]	40	ANN	Automatic heart defect detection for athletes	Accuracy = 0.9
Adetiba et al. [25]	40	ANN	Develop a wearable ECG that can be worn by athletes to help automatically detect defects	Accuracy = 1
Barbieri et al. [34]	26,002	Decision trees Logistic regression	Classify whether an athlete is at cardiovascular risk or not	AUC = 0.78
Bernardino et al. [36]	-	Logistic regression Principal component analysis Statistical shape analysis	Highlight areas of the heart that undergo cardiac remodelling due to endurance exercise	-
Castillo-Atroche et al. [37]	56,542 samples from 487 patients	CNN	Automatically predict arrhythmias in athletes in real time	Accuracy = 0.939
Christ and Rückert [40]	22 and 9	ANN Random forest Support vector machine	Predict whether a patient was an athlete or not based on ECG readings	Accuracy = 0.981
Długosz et al. [16]	160	Decision tree Logistic regression	(1) Use ECGs to estimate the level of cardiac troponin (cTnI) in amateur athletes (2) Detect coronary artery disease (CAD) in athletes	AUC = 0.91
Huang et al. [19]	598	Agglomerative hierarchical Clustering Multiple regression analysis	(1) Identify athlete groups with similar characteristics (2) Investigate the validity of sport-specific adaption for evaluating athlete’s hearts	-
Hussain at al [20]	7200 data points from 4 athletes	LSTM	(1) Predict and athlete’s health state (2) Predict the activity being performed by an athlete	(1) Accuracy = 0.97 (2) Accuracy = 0.83
Laurino et al. [21]	14 and 12	ANN K nearest neighbours Naïve Bayes Support vector machines	Classifying heart states in athletes between those at rest and those in stressful conditions	Accuracy = 0.86
Lombardi et al. [22]	26	Linear discriminant analysis	Determine whether patients with idiopathic ventricular arrhythmias with left bundle branch block and inferior axis morphology arrhythmia originated from the aortic sinus cusps or the right ventricular outflow tract	Accuracy = 0.947
Narula et al. [26]	139	ANN Random forest Support vector machine	Discriminate between hypertrophic cardiomyopathy from physiological hypertrophy in athletes	AUC = 0.795
Rahmen et al. [27]	470	Naïve Bayes Random forest Support vector machines	Predict whether an athlete’s heart is normal or not	Accuracy 0.742 and 0.553 for experiments 1 and 2, respectively
